# Electroconvulsive therapy for obsessive-compulsive disorder: clinical efficacy and predictive role of inflammatory markers

**DOI:** 10.3389/fpsyt.2025.1715406

**Published:** 2026-01-29

**Authors:** Shengkang Hu, Chenchen Zhang, Rui Li, Cuiyuan Fu, Bin Wang, Kun Li

**Affiliations:** 1 Shandong Daizhuang Hospital, Jining, Shandong, China; 2 Jining Key Laboratory of Neuromodulation, Jining, Shandong, China

**Keywords:** clinical efficacy, electroconvulsive therapy, inflammatory markers, obsessive-compulsive disorder, seizure duration

## Abstract

**Objective:**

This retrospective study aimed to evaluate the clinical efficacy of electroconvulsive therapy (ECT) in patients with obsessive-compulsive disorder (OCD) and identify potential predictive factors, focusing on inflammatory markers to optimize therapeutic outcomes.

**Methods:**

We retrospectively analyzed clinical data from 63 OCD patients who received ECT from January 2010 to August 2024. Clinical demographics, ECT parameters, and baseline blood cell counts were extracted from electronic medical records. Inflammatory indices were calculated accordingly. The Clinical Global Impressions-Improvement scale (CGI-I) was utilized to assess treatment outcomes. Patients were categorized into responders and non-responders based on CGI-I scores. Logistic regression analysis determined predictors of ECT efficacy, and the receiver operating characteristic (ROC) curve assessed the predictive capability of these factors.

**Results:**

Among the 63 patients, 46 (73.0%) responded positively to ECT. Responders had significantly more ECT sessions (*Z* = - 3.03, *P* = 0.002), longer seizure durations (*Z* = -2.40, *P* = 0.016), and higher baseline neutrophil-to-monocyte ratio (NMR) values (*T* = 2.76, *P* = 0.008) compared to non-responders. Logistic regression analysis demonstrated seizure duration was independently associated with efficacy (*OR* = 1.206, *P* = 0.019), while baseline NMR did not achieve statistical significance in the multivariate analysis (*OR* = 1.264, *P* = 0.063). ROC analysis indicated that seizure duration has significant predictive capability (*AUC* = 0.696; sensitivity 63.0%, specificity 71.6%).

**Conclusion:**

ECT is clinically effective for OCD, seizure duration was significantly associated with treatment outcomes. Although baseline NMR was not statistically significant in multivariate analysis, it holds potential predictive value, possibly limited by sample size and study design. Future larger-scale, prospective studies are warranted.

## 1 Introduction

The widespread mental illness known as obsessive-compulsive disorder (OCD) is typified by recurring, intrusive thoughts (called obsessions) and repetitive actions (called compulsions), and it is a common mental disorder. The lifetime prevalence of OCD is as high as 2-3% (1). Due to the unclear pathophysiological mechanisms, effective treatments are still lacking. Selective serotonin reuptake inhibitors (SSRIs) and exposure and response prevention (ERP) therapy are recognized as first-line treatments (2); however, these methods yield variable efficacy, 40%-60% of patients show either no response, only partial response, or even develop resistance to first-line drug treatments (3, 4). Therefore, it is necessary to develop new evidence-based strategies for treating OCD patients.

New pharmacological treatments, non-invasive and invasive neurostimulation techniques, are continuously evolving and have been applied to the treatment of refractory OCD (5). However, the evidence base for these new therapies is limited. The clinical efficacy of invasive neurostimulation therapies such as deep brain stimulation (DBS) for treating OCD has been demonstrated (6). However, its widespread application is limited due to the invasiveness of the procedure, the requirement for general anesthesia, high costs, and associated risks (7).

Electroconvulsive therapy (ECT), a well-established treatment involving the induction of seizures via electric currents applied to the brain, has demonstrated considerable effectiveness and safety in various severe psychiatric disorders, including major depressive disorder, schizophrenia, and OCD (8–10). Previous studies evaluating the efficacy of ECT in refractory OCD patients have shown significant reductions in obsessive-compulsive symptoms, depression, and anxiety scores compared with medication-only treatments (11).

In recent years, the role of neuroinflammation in the pathophysiology of OCD has gained increasing attention. Substantial evidence indicates that mild inflammation and a predisposition toward heightened inflammatory responses are prevalent in the early lives of individuals with OCD (12). More importantly, during neurodevelopmental windows, a balanced neuroinflammatory process is crucial for proper neural circuit formation, establishment of neuronal connectivity, neurogenesis, and neuronal survival, mediated by microglial synaptic pruning (13–16). It is thus hypothesized that disruption of this balanced neuron-microglia interaction may lead to an exacerbated inflammatory process both spatially and temporally, potentially triggering a range of neural and behavioral abnormalities, including OCD (17). Notably, ECT has been demonstrated to act as an effective neuroimmunomodulator. Beyond its regulatory effects on neurotransmitters and neurotrophic factors, studies show that ECT directly targets the innate immune system within the central nervous system, alleviating neuroinflammation by reducing microglial cytotoxicity (18). Following both acute and chronic ECT, neuroinflammatory responses gradually diminish over time, while microglial activation and neurogenesis are sustained (19). Therefore, we speculate that for OCD patients with a specific background of immune dysregulation, ECT may exert its therapeutic effects by “calibrating” their abnormal immune status. Based on this, peripheral blood inflammatory markers are expected to serve as practical tools for identifying such “inflammatory subtype” patients who may respond better to ECT.

Given the promising yet limited evidence on ECT’s effectiveness for OCD, and the predictors of treatment response remain unclear, clinical decision-making lacks precision. Current studies are mostly confined to reporting efficacy and lack systematic evaluation of treatment parameters and easily accessible biomarkers. This study aims to combine treatment parameters (such as seizure duration) and biomarkers (such as inflammatory markers) in a retrospective analysis to construct a comprehensive predictive model. Therefore, we propose the research hypothesis: longer seizure duration and elevated baseline inflammatory marker levels can independently predict a positive treatment response to ECT in patients with OCD.

## 2 Materials and methods

### 2.1 Study design and population

This study included OCD patients who were hospitalized and received ECT treatment at Shandong Daizhuang Hospital from January 2010 to August 2024. In our hospital, the use of ECT for OCD patients follows explicit criteria based on international guidelines and local protocols. Patients must meet at least one of the following criteria (1): Pharmacotherapy resistance: Defined as an inadequate response to an adequate dose and duration of at least two trials of antidepressants from different pharmacological classes (2); Severe self-harm or suicidality: Defined as the presence of active, imminent suicidal ideation with a specific plan or a recent (within one month) suicide attempt; (3) Poor medication adherence: The patient lacks insight, has no awareness of their illness, and does not believe they are sick; (4) Impulsive behavior: The patient displays significant impulsive and aggressive behaviors that are difficult to manage with medication. For the enrolled patients, we established strict inclusion and exclusion criteria. Inclusion Criteria: (1) Aged 18–65 years; (2) Primary diagnosis of OCD according to the International Classification of Diseases (ICD-10); (3) Meeting at least one of the ECT indication criteria listed above; (4) Underwent at least one ECT session at our hospital. Exclusion Criteria: (1) Contraindications to ECT (e.g., increased intracranial pressure, recent myocardial infarction); (2) Comorbid other primary psychiatric disorders (e.g., schizophrenia, current manic episode of bipolar disorder); (3) Presence of severe, unstable physical illness; (4) Pregnancy or lactation; (5) Presence of an autoimmune disease; (6) Acute infection within 4 weeks prior to or during the ECT course; (7) Use of anti-inflammatory or corticosteroid medications within 4 weeks prior to or during ECT.

**Figure 1:**
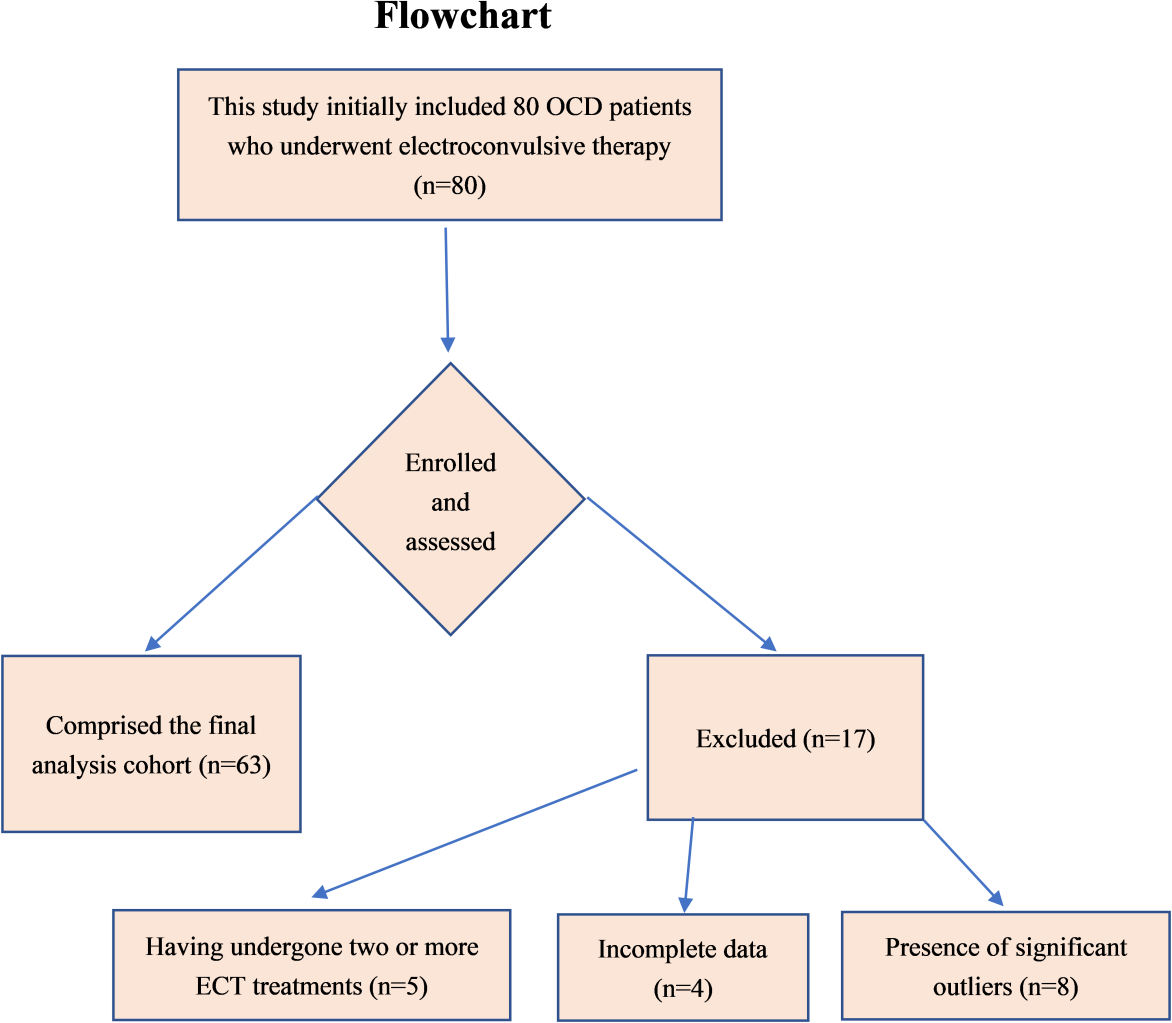
Flowchart.

A total of 80 OCD patients were initially included in this study. Among them, 5 patients had undergone two or more ECT sessions, and only data from their first ECT treatment were included in the analysis. Following data review and preliminary analysis, 12 patients were excluded due to incomplete data or the presence of significant outliers. Of these 12 patients, 4 had missing baseline blood cell counts, while 8 exhibited significant outliers in either baseline blood cell counts or ECT parameters. Ultimately, 63 OCD patients were included in the study (see **Figure 1**). A comparative analysis of baseline characteristics revealed that the included group was significantly younger in terms of both age and age of onset compared to the excluded group (*P* < 0.05).

This study obtained ethical approval from the Ethics Committee of Shandong Daizhuang Hospital (Approval No. 2024-No.58-202410HM-1) and was carried out in compliance with the principles outlined in the Declaration of Helsinki. As the research qualified for institutional review exemption, informed consent was not required. Additionally, the trial has been officially registered with the Chinese Clinical Trial Registry under the registration number ChiCTR2400093247.

### 2.2 Electroconvulsive therapy protocol

A THYMATRON System IV device from Somatics, LLC, located in Lake Bluff, IL, USA, was used to administer electroconvulsive therapy. Treatment plans, including potential benefits and risks, were discussed comprehensively with patients and their legal guardians. Signed informed consent must be obtained prior to the initiation of treatment. Each patient’s clinical indications and contraindications were reviewed by a multidisciplinary medical team before initiating ECT.

ECT sessions were typically scheduled three times weekly on alternate days. Bilateral electrode placement on the temporal regions was employed. Procedures were conducted under general anesthesia induced by propofol and muscle relaxation achieved with succinylcholine, supervised by anesthesiologists and psychiatrists. During the ECT procedure, the propofol dose was fixed at 90 mg, the time interval from the completion of the final propofol bolus to the application of electrical stimulation was strictly controlled within the range of 2–3 minutes. Data collected included the total number of ECT sessions, duration of electrical stimulation, seizure duration, delivered electrical charge, and stimulus energy.

In this study, the ECT device was not connected to an electroencephalogram (EEG). Seizure duration was measured by observing motor manifestations using the “cuff method” (20). Prior to the administration of the muscle relaxant (succinylcholine), a blood pressure cuff was inflated on one of the patient’s ankles to isolate that limb. The seizure duration was then timed from the beginning to the end of the tonic-clonic motor activity observed in that isolated limb. Regarding treatment parameters, particularly stimulus dose, the treatment protocol was determined through individualized titration based on each patient’s specific characteristics.

### 2.3 Clinical assessments and data collection

Clinical response was evaluated using the Clinical Global Impressions-Improvement scale (CGI-I) (21). Based on common practices in the field and with a focus on identifying clinically meaningful improvements (22). Patients were classified as responders (CGI-I scores of 1–2, indicating significant improvement) or non-responders (CGI-I scores of 3–4, indicating minimal or no improvement). This dichotomization ensures that the ‘responder’ category represents a clear, clinically relevant positive outcome and facilitates robust statistical comparisons given the available sample size.

Demographic and clinical data, including gender, age, age of onset, education level, illness duration, and reasons for initiating ECT (treatment resistance, suicidal ideation, poor medication adherence, impulsivity), were extracted from electronic medical records. Baseline blood tests prior to ECT recorded platelet counts, absolute counts of neutrophils, lymphocytes, monocytes, basophils, and eosinophils. Inflammatory markers, including platelet-to-lymphocyte ratio (PLR), neutrophil-to-lymphocyte Ratio (NLR), monocyte-to-lymphocyte Ratio (MLR), neutrophil-to-monocyte ratio (NMR), and systemic immune-inflammation index (SII), were calculated accordingly (23, 24).

### 2.4 Statistical analyses

Data analyses were conducted using IBM SPSS Statistics version 26.0 and R4.5.2. Descriptive statistics were generated for demographic, clinical, and treatment characteristics, reporting means, standard deviations, medians, and frequency distributions as appropriate. Normality of the data was tested using the Shapiro-Wilk test, the choice of analytical methods depends on whether the data conform to a normal distribution. Between-group comparisons were performed using independent samples t-tests or Mann-Whitney *U* tests for continuous variables and chi-square or Fisher’s exact tests for categorical variables. To explore the potential reverse causality bias between the number of ECT sessions and treatment response, we conducted a landmark analysis. This analysis was restricted to those patients who had completed at least 6 ECT sessions — a common threshold in ECT practice (25).

Variables found to be significant through univariate analysis were subsequently included in a multivariate logistic regression model to determine independent predictors of ECT efficacy. Multicollinearity and potential outliers were evaluated prior to model fitting. The predictive capability of significant factors was further evaluated through receiver operating characteristic (ROC) curve analysis. To comprehensively assess the model’s performance, we evaluated its calibration using a calibration plot and the Hosmer-Lemeshow test. Internal validation was performed via 1000 bootstrap samples to quantify any overfitting and to obtain a bias-corrected area under the curve (AUC). The clinical utility of the predictive model was quantified using decision curve analysis (DCA), which estimates the net benefit across a range of threshold probabilities. Furthermore, for the optimal cut-off value identified by the ROC analysis, positive predictive value (PPV) and negative predictive value (NPV) were calculated to enhance clinical interpretability. Statistical significance was established at a threshold of *P* < 0.05.

## 3 Result

### 3.1 Demographic and clinical characteristics

As summarized in **Table 1**, an analysis was conducted on a cohort of 63 patients diagnosed with OCD who received ECT. Among these patients, 40 individuals (63.5%) were male, while 23 individuals (36.5%) were female. The mean age was 27.65 ± 9.62 years. Regarding education, 50.7% had completed high school education or above.

**Table 1:** Comparison of clinical and ECT characteristics between response and non-response groups.

Variable	Total (n=63)	Response group (n=46)	Non-response group (n=17)	*Z*, *T* or *χ²*	*P* value
Age (years)	27.65 ± 9.62	28.43 ± 9.85	25.53 ± 8.88	-1.14	0.254
Education Level				2.97	0.400
Primary school	10 (15.9%)	5 (15.2%)	3 (17.6%)		
Junior high school	21 (33.3%)	15 (32.6%)	6 (35.3%)		
High school	20 (31.7%)	17 (37.0%)	3 (17.6%)		
College	12 (19.0%)	7 (15.2%)	5 (29.4%)		
Gender				0.51	0.477
Male	40 (63.5%)	28 (60.9%)	12 (70.6%)		
Female	23 (36.5%)	18 (39.1%)	5 (29.4%)		
Age of onset (years)	20.25 ± 7.63	20.41 ± 7.95	19.82 ± 6.89	-0.37	0.709
Illness duration (months)	88.79 ± 81.45	96.35 ± 83.77	68.35 ± 73.23	-1.47	0.142
Reasons for ECT				1.42	0.834
Treatment resistance	56 (88.9%)	39 (84.8%)	14 (82.4%)		
Suicidal ideation	8 (12.7%)	3 (6.5%)	2 (11.8%)		
Poor medication adherence	2 (3.2%)	2 (4.3%)	0 (0)		
Impulsivity	3 (4.8%)	2 (4.3%)	1 (5.9%)		
Electrical parameters					
Number of sessions	10.24 ± 3.66	10.98 ± 3.47	8.24 ± 3.51	-3.03	0.002
Stimulus duration	5.44 ± 1.67	5.26 ± 1.83	5.93 ± 1.07	-1.05	0.292
Seizure duration (s)	18.33 ± 6.74	19.63 ± 7.10	14.82 ± 4.08	-2.40	0.016
Energy (J)	26.15 ± 9.57	26.95 ± 10.24	24.00 ± 7.30	-0.79	0.430
Side effects				4.34	0.110
None	51 (81.0%)	39 (84.8%)	12 (70.6%)		
Head discomfort	9 (14.3%)	4 (8.7%)	5 (29.4%)		
Amnesia	3 (4.8%)	3 (6.5%)	0 (0)		
Inflammatory indicators					
PLR	126.90 ± 50.67	129.02 ± 56.67	121.17 ± 29.54	0	1
MLR	0.23 ± 0.14	0.24 ± 0.17	0.22 ± 0.06	-0.17	0.865
NLR	2.13 ± 2.09	2.30 ± 2.40	1.66 ± 0.60	-1.61	0.107
NMR	9.14 ± 3.11	9.64 ± 3.33	7.78 ± 1.91	2.76	0.008
SII	519.16 ± 612.24	565.00 ± 707.58	395.12 ± 148.36	-1.21	0.227

All variables are presented as “mean ± standard deviation” or “number (percentage)”. OCD, obsessive-compulsive disorder; ECT, electroconvulsive therapy; PLR, platelet-to-lymphocyte ratio; MLR, monocyte-to-lymphocyte ratio; NLR, neutrophil-to-lymphocyte ratio; NMR, neutrophil-to-monocyte ratio; SII, systemic immune-inflammation index.

Regarding clinical information, the average illness duration was 88.79 ± 81.45 months, and the average age of onset was 20.25 ± 7.63 years. Treatment resistance was the most common indication for ECT, noted in 88.9% of patients.

The overall response rate to ECT treatment was 73.0%. The mean number of ECT sessions was 10.24 ± 3.66. Most patients (81.0%) reported no adverse effects; however, some reported minor issues such as headaches (14.3%) and memory impairment (4.8%) (see **Table 1** for details).

### 3.2 Comparison between responders and non-responders

A total of 63 patients were included in the study, with 46 (73.0%) categorized as responders and 17 (27.0%) as non-responders, as shown in **Table 1**. There were no statistically significant differences between the two groups with regard to demographic characteristics, including age (*Z* = -1.14, *P* = 0.254), gender (*χ²* = 0.51, *P* = 0.477) and education level (*χ²* = 2.97, *P* = 0.400). Regarding clinical characteristics, no significant differences were found in age of onset (*Z* = -0.37, *P* = 0.709), illness duration (*Z* = -1.47, *P* = 0.142), or the reasons for ECT between the two groups (*χ²* = 1.42, *P* = 0.834).

In terms of ECT parameters, responders received significantly more ECT sessions (*Z* = -3.03, *P* = 0.002) and had longer seizure durations (*Z* = -2.40, *P* = 0.016) in comparison to non-responders. However, there were no significant differences in the energy delivered during treatment between the two groups (*Z* = -0.79, *P* = 0.430). The landmark analysis results show that among the total of 63 patients, 53 had received at least six ECT sessions. Among these, responders (n=40) received a mean of 12.08 ± 2.08 sessions, while non-responders (n=13) received a mean of 9.62 ± 2.66 sessions. The difference between the two groups remained statistically significant (*Z* = -3.240, *P* = 0.001).

For inflammatory markers, no significant differences were found between the groups in PLR (*Z* = 0.00, *P* = 1.000), MLR (*Z* = -0.17, *P* = 0.865), SII (*Z* = -1.21, *P* = 0.227), and NLR (*Z* = -1.61, *P* = 0.107). Nevertheless, the NMR was markedly elevated in the responder group relative to the non-responder group (*T* = 2.76, *P* = 0.008).

### 3.3 Predictive factors for ECT efficacy

To identify predictors of ECT efficacy, variables with significant differences between responders and non-responders were entered into a logistic regression analysis, including seizure duration and NMR. As shown in **Table 2**, the results revealed that seizure duration was an independent associated with efficacy (*OR* = 1.206, *P* = 0.019), indicating that longer seizure durations were associated with a higher likelihood of a positive treatment response. However, baseline NMR did not achieve statistical significance in the multivariate analysis (*OR* = 1.264, *P* = 0.063). There is no severe multicollinearity and outliers between the two variables. Although the number of treatment sessions showed a significant difference between the two groups, it was not included in the logistic regression analysis as it is not a baseline variable.

**Table 2:** Logistic regression analysis of factors predicting the efficacy of electroconvulsive therapy for obsessive-compulsive disorder.

Variable	*β*	SE	Wald*χ²*	*OR*	95%CI	*P*-value
Seizure duration	0.187	0.080	5.466	1.206	1.031-1.411	0.019
NMR	0.234	0.126	3.445	1.264	0.987-1.618	0.063

NMR, neutrophil-to-monocyte ratio.

**Figure 2:**
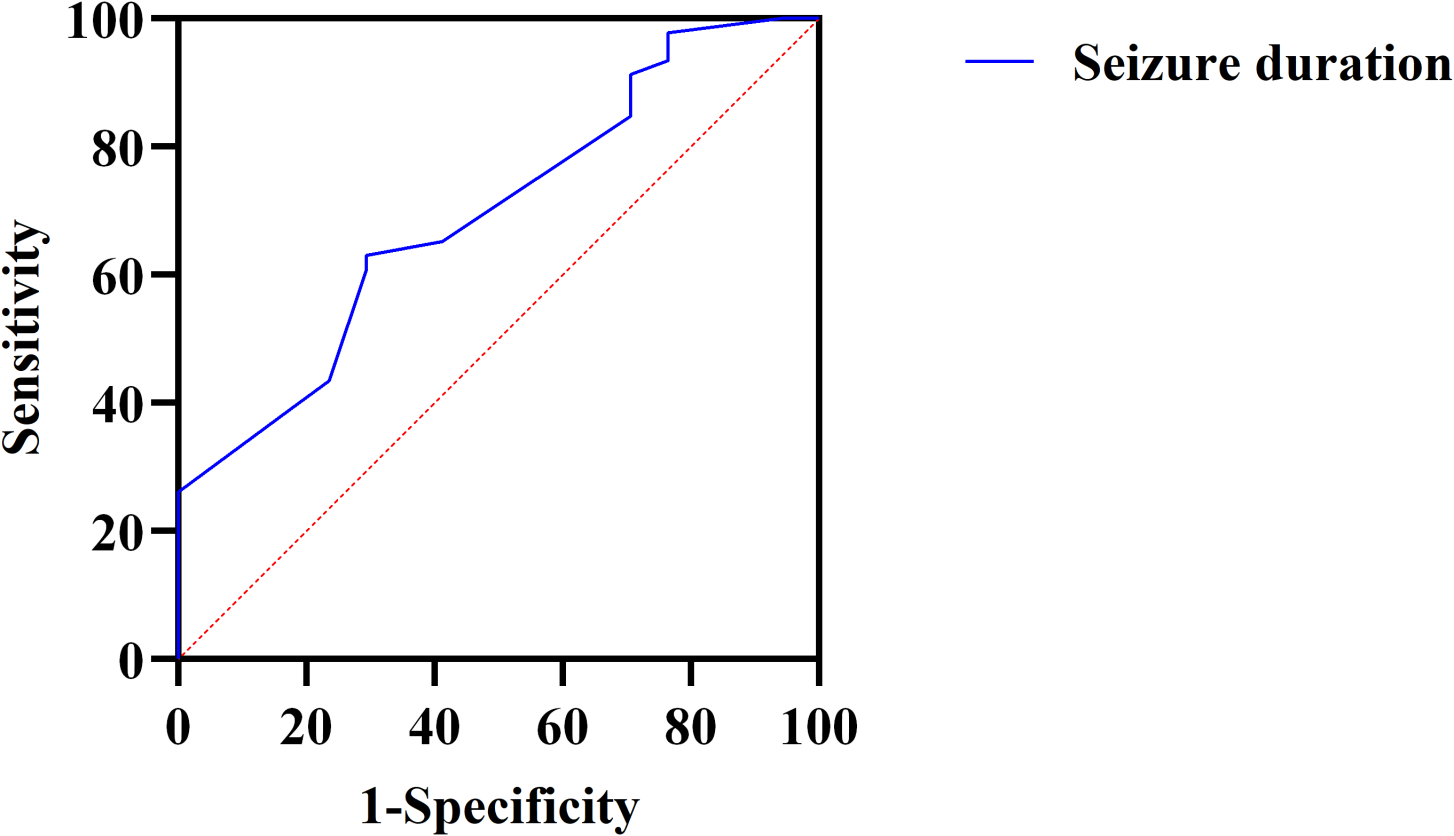
ROC curve for predicting the efficacy of ECT based on seizure duration in patients with OCD. ROC, Receiver Operating Characteristic; ECT, electroconvulsive therapy; OCD, obsessive-compulsive disorder.

ROC curve analysis was conducted to assess the predictive ability of seizure duration for treatment efficacy. The area under the curve (AUC) was 0.696, indicating moderate predictive power. The optimal cut-off value for seizure duration was determined to be 16.50 seconds, with a sensitivity of 63.0% and specificity of 71.6%, suggesting that seizure duration has a reasonable ability to predict the likelihood of a favorable treatment response (**Figure 2**).

**Figure 3:**
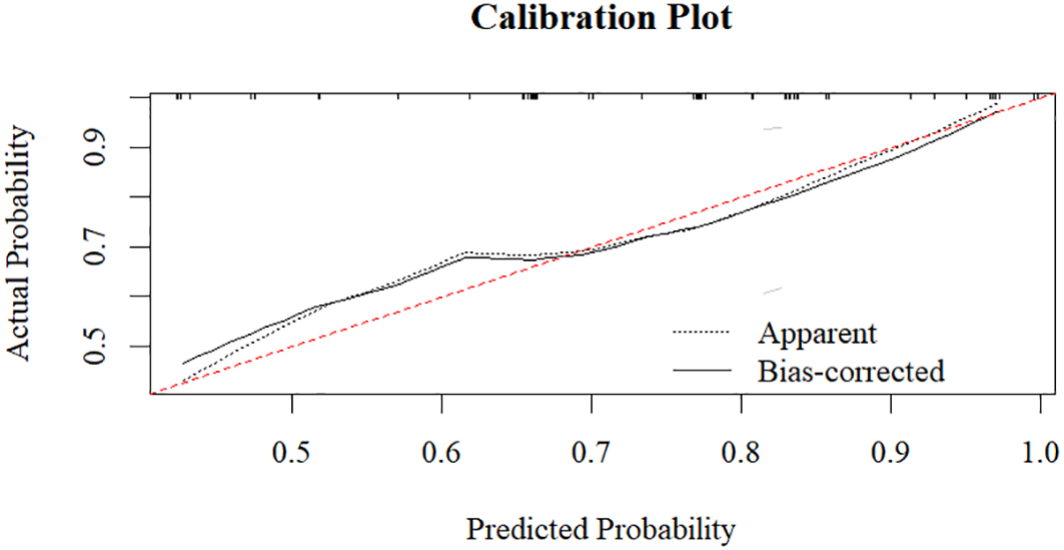
Calibration plot of the seizure duration model.

**Figure 4:**
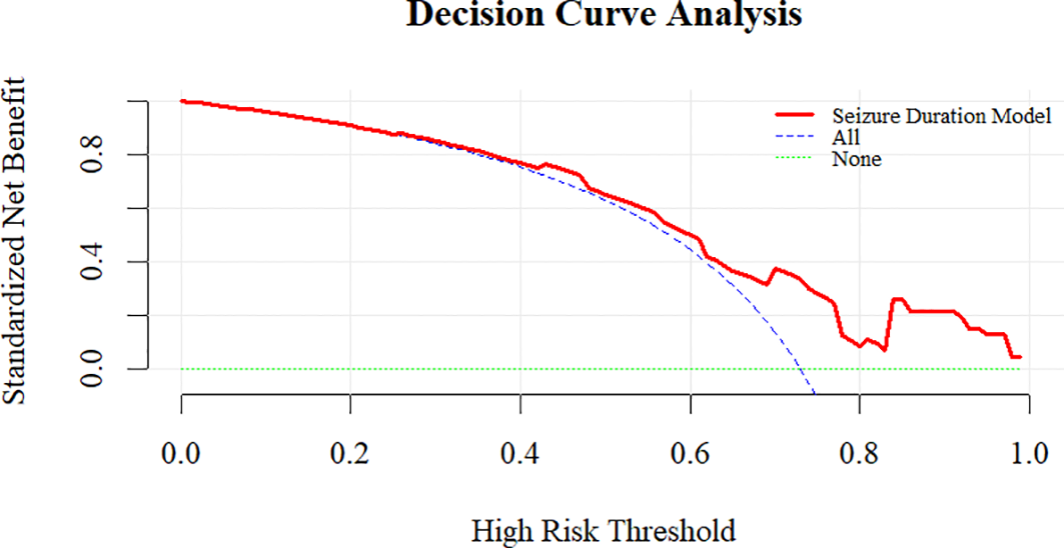
Decision curve analysis evaluating the net clinical benefit of the seizure duration model in predicting ECT response.

To further validate the predictive model, we performed additional evaluations. The calibration plot demonstrated good agreement between predicted and observed probabilities of ECT response (Hosmer-Lemeshow test, *P* = 0.543) (**Figure 3**). Internal validation with 1000 bootstrap resamples revealed an optimism-corrected AUC of 0.696, indicating minimal overfitting. Decision curve analysis demonstrates that the prediction model shows favorable net benefit across a broad threshold probability range of 0 to 0.7 (**Figure 4**). Specifically, the net benefit reaches its peak at thresholds below 0.4, this indicates that using the model to guide clinical decisions is superior to strategies of “treat all” or “treat none”. At the optimal seizure duration cut-off of 16.50 seconds, the positive predictive value (PPV) was 85.3%, and the negative predictive value (NPV) was 41.4%.

## 4 Discussion

In this study, we found that the overall efficacy rate of ECT for OCD was 73.0%. Compared to non-responders, responders received more ECT sessions, had longer seizure durations, and exhibited higher baseline Neutrophil-to-Monocyte Ratio (NMR) values. These findings are consistent with previous research, though some differences exist due to sample size and study design.

The response rate observed in this study aligns with previous reports, which also suggest a positive response to ECT in refractory OCD (26). In a systematic review of ECT for OCD, the overall positive response rate was reported at 60.4% (27), which is somewhat lower than our findings, though this may be attr ibuted to the inclusion of a broader range of studies, many of which were case reports. Other reviews have reported a 73.0% positive response rate, supporting the clinical utility of ECT for OCD (28). This implies that ECT can give rise to significant improvements in obsessive-compulsive symptoms to some extent (29–31). However, the current evidence substantiating the clinical efficacy of ECT in treating OCD remains insufficient. Further experimental cohort studies are needed to confirm its efficacy rate more conclusively.

Interestingly, we observed that responders to ECT tended to receive more treatment sessions, a finding that could be linked to the effectiveness of repeated ECT sessions in regulating neurotransmitters and neuroplasticity. Some researchers speculate that effects of ECT on the serotonin system and brain-derived neurotrophic factor (BDNF) levels may explain this association (32, 33). Elevated levels of BDNF following ECT treatment are thought to promote neurogenesis and improve neuronal function, which might contribute to the observed clinical improvements. Additionally, previous studies have suggested that the cumulative effects of multiple ECT sessions are critical for achieving significant symptom relief (34, 35). Our study supports this notion, suggesting that the number of sessions serves a role in determining the therapeutic outcome of ECT in OCD patients. It is also essential to consider alternative explanations for the finding of this study: Although we observed that responders received more ECT sessions, reverse causality remains a plausible concern. Therefore, we performed a landmark analysis, the results of which provide important context for interpreting the role of treatment sessions. This analysis, which restricted the cohort to patients who had completed at least six sessions, demonstrated that responders still received a significantly greater mean number of sessions than non-responders. Since this analysis effectively aligns the sample at a common early timepoint in the treatment course, it mitigates the concern that the observed association was solely due to non-responders discontinuing treatment early because of a lack of efficacy. While a randomized controlled trial would be required to definitively establish causality, this finding strengthens the inference that undergoing a more extensive ECT course is associated with a higher likelihood of a positive response, supporting the clinical practice of ensuring an adequate number of treatments for OCD patients undergoing ECT. Prospective studies are needed in the future to clarify the potential causal dynamics.

Responders showed higher values across several peripheral inflammatory markers; among these, NMR differed significantly between groups, though it did not retain independent predictive value in multivariable models, which suggests a potential association between the baseline inflammatory state and ECT treatment outcomes. An immuno-inflammatory framework for OCD has gained traction (36, 37), positing that low-grade peripheral inflammation can alter blood–brain barrier dynamics, activate microglia, and trigger cascades affecting neurotransmission and plasticity (38–41). ECT may act, in part, by modulating neuroimmune pathways—promoting hippocampal gliogenesis/neurogenesis (42, 43), altering microglial activity, and increasing neurotrophin levels (44–47), alongside transmitter-system recalibration (48). In depression and schizophrenia, higher baseline inflammatory burden has been linked to better ECT outcomes in some studies (49–51). By extension, NMR could serve as a pragmatic proxy of “inflammatory susceptibility, “identifying patients likelier to benefit from ECT-induced neuroimmune modulation. The interpretation of these findings requires caution and should consider the following possibilities. As a peripheral marker measured only at baseline, the NMR may not fully capture the central neuroimmune dynamics crucial to OCD or the evolving immunomodulatory effects of ECT. Its predictive value might be enhanced by tracking its dynamic changes and integrating it with other biomarkers. Future prospective studies should integrate multi-timepoint and multidimensional biomarkers to establish more robust predictive models of treatment response and to clarify the immunomodulatory mechanisms underlying ECT in OCD.

Seizures duration has long been considered a crucial parameter for evaluating the effectiveness of ECT since it is simple to measure through movement or electroencephalograms (EEGs). In our study, the seizure duration is a factor affecting the efficacy of ECT in patients with OCD. The same finding has been confirmed in studies of individuals with MDD, where seizure duration is associated with remission of MDD, and shorter seizure duration is correlated with a lower remission rate after ECT (52). The American Psychiatric Association guidelines recommend a minimum threshold of 15 seconds for either motor or electrical seizure activity as the efficacy criterion (53). Previous studies have suggested that the total seizure duration during ECT treatment is correlated with therapeutic response. A total seizure duration of less than 210 seconds predicts ineffectiveness in response to ECT treatment, and therapeutic outcomes will significantly improve when the duration reaches 300 seconds or higher (54). It can be inferred that seizure duration has an important impact on the therapeutic efficacy of ECT. However, given the exploratory and retrospective nature of our study, there is uncertainty as to whether there is a causal relationship between seizure duration and clinical outcomes. It is plausible that longer seizure durations may not solely be a cause of favorable treatment response, but could also, in part, be a consequence thereof. This potential bidirectional relationship implies that seizure duration could partly function as a marker of the ongoing therapeutic process, rather than purely a baseline predictor. A seizure duration of ≥ 16.5 seconds” is a robust, confidence-building predictive rule; however, it should not be used to rule out the possibility of a successful treatment. Because seizure duration is influenced by stimulus dosing, electrode placement, anesthetic and muscle relaxant regimens, age, and concomitant medications (55, 56), clinicians can optimize this parameter through individualized titration and technical refinements. Still, prolonging seizures should not be pursued in isolation; comprehensive quality indices—such as ictal morphology and post-ictal suppression—remain integral to safe, effective care. Well-designed prospective studies are needed in the future to specifically analyze the relationship between the duration of seizures from the initial treatment phase and their long-term outcomes, in order to clarify the temporal sequence and better establish its predictive utility.

In this study, we adopted a dichotomized CGI-I score to evaluate treatment efficacy, based on the consideration that “response” should represent unequivocal clinical benefit. It is noteworthy that within the non-responder group, some patients had a CGI-I score of 3 (“minimally improved”). This subgroup constitutes an interesting “borderline” or “threshold” cohort. Their presence suggests that the therapeutic effect of ECT may exist on a continuum rather than being an all-or-none phenomenon. The suboptimal response in these minimally improved patients could be attributable to factors such as an incomplete treatment course, suboptimal individualized stimulation parameters, or the possibility that they represent a distinct clinical or biological subtype with a different pattern of response to ECT. Future studies with larger sample sizes could analyze these “partial responders” as a separate category, which would help refine our understanding of the ECT efficacy spectrum and explore strategies to elevate their improvement from “minimal” to “significant.”

This single-center, retrospective study is susceptible to selection bias and residual confounding. First, regarding selection bias, the study has confirmed that the exclusion of cases due to incomplete data may have introduced selection bias, as the excluded patients were older and had a later age of onset, which may limit generalizability. To verify robustness, we repeated our logistic regression including these as covariates. Seizure duration remains an independent influencing factor of ECT efficacy (OR = 1.207, p=0.019), confirming our core finding was not confounded. However, a further important limitation is the potential for residual confounding by unmeasured variables. Most notably, detailed data on specific psychiatric comorbidities (such as co-occurring depressive or anxiety disorders) and concurrent psychotropic medications (such as types and dosages of antidepressants or antipsychotics) were not systematically available for adjustment in our analysis. These factors are known to influence both the clinical profile of OCD and potentially the response to ECT, and their absence represents a limitation of the present study.

The inpatient, clinically severe sample constrains generalizability. Outcomes were rated with CGI-I rather than a standardized Y-BOCS, reducing symptom-domain granularity. Our choice of CGI-I was pre-defined to assess global clinical response in a real-world setting, as it captures overall functional improvement, including comorbidities and quality of life. We would like to clarify that literature supports the validity of CGI-I in OCD trials, demonstrating significant correlation with Y-BOCS scores (57). The measurement of seizure duration relied on the cuff-based motor observation method rather than EEG. Consequently, we were unable to assess more precise electrophysiological correlates of seizure adequacy, such as ictal EEG amplitude or postictal suppression, which are considered important qualitative indicators of ECT treatment. There may be a bidirectional causal relationship between seizure duration and treatment efficacy, which could compromise the independent predictive power of this indices.

Given these methodological limitations, future studies should employ prospective, multi-center designs with consecutive patient enrollment to enhance generalizability. The objective measurement of seizure quality using EEG and the application of standardized efficacy scales are warranted. Furthermore, analytical methods such as landmark analysis should be considered to better address potential reverse causality.

## 5 Conclusion

Our findings reinforce the clinical effectiveness of ECT for OCD and identify seizure duration as an independent factor significantly associated with treatment response. Baseline NMR shows preliminary promise as an adjunctive stratification tool but requires higher-quality evidence. A combined strategy—adequate course completion, optimization of ictal quality, and inflammation-informed stratification—may advance the individualized use of ECT in OCD.

## Data Availability

The raw data supporting the conclusions of this article will be made available by the authors, without undue reservation.
